# High-Resolution Linkage Analyses to Identify Genes That Influence *Varroa* Sensitive Hygiene Behavior in Honey Bees

**DOI:** 10.1371/journal.pone.0048276

**Published:** 2012-11-02

**Authors:** Jennifer M. Tsuruda, Jeffrey W. Harris, Lanie Bourgeois, Robert G. Danka, Greg J. Hunt

**Affiliations:** 1 Department of Entomology, Purdue University, West Lafayette, Indiana, United States of America; 2 Honey Bee Breeding, Genetics and Physiology Laboratory, United States Department of Agriculture – Agricultural Research Service, Baton Rouge, Louisiana, United States of America; Arizona State University, United States of America

## Abstract

*Varroa* mites (*V. destructor*) are a major threat to honey bees (*Apis melilfera*) and beekeeping worldwide and likely lead to colony decline if colonies are not treated. Most treatments involve chemical control of the mites; however, *Varroa* has evolved resistance to many of these miticides, leaving beekeepers with a limited number of alternatives. A non-chemical control method is highly desirable for numerous reasons including lack of chemical residues and decreased likelihood of resistance. *Varroa* sensitive hygiene behavior is one of two behaviors identified that are most important for controlling the growth of *Varroa* populations in bee hives. To identify genes influencing this trait, a study was conducted to map quantitative trait loci (QTL). Individual workers of a backcross family were observed and evaluated for their VSH behavior in a mite-infested observation hive. Bees that uncapped or removed pupae were identified. The genotypes for 1,340 informative single nucleotide polymorphisms were used to construct a high-resolution genetic map and interval mapping was used to analyze the association of the genotypes with the performance of *Varroa* sensitive hygiene. We identified one major QTL on chromosome 9 (LOD score = 3.21) and a suggestive QTL on chromosome 1 (LOD = 1.95). The QTL confidence interval on chromosome 9 contains the gene ‘no receptor potential A’ and a dopamine receptor. ‘No receptor potential A’ is involved in vision and olfaction in *Drosophila*, and dopamine signaling has been previously shown to be required for aversive olfactory learning in honey bees, which is probably necessary for identifying mites within brood cells. Further studies on these candidate genes may allow for breeding bees with this trait using marker-assisted selection.

## Introduction

Pollination by honey bees (*Apis mellifera*) is an important part of modern agriculture, and honey bee health has been receiving increased attention recently from the public, beekeepers, and researchers. Honey bees face numerous challenges, including pesticides, pathogens, and parasites (such as *Varroa* mites, *V. destructor*) [1]–[3]. *Varroa* parasitism of honey bees is widely considered to be the greatest threat to beekeeping and has led to substantial colony losses worldwide [4]–[10]. These obligate ectoparasites live in the nest of honey bees and harm individuals and colonies.

The mites require developing honey bees for their reproduction. Mated adult female mites enter brood cells and start laying eggs, one male and up to five female in worker brood cells but an average of 1.3–1.45 new, mature female offspring are produced [11], [12]. The offspring feed on the hemolymph of the developing bee pupa and sibling mites mate with one another. When the adult bee emerges, mature female mites leave the worker cell and enter a phoretic stage while feeding on the hemolymph of adult bees [13]. The cycle is repeated when the female mite enters a new brood cell. When *Varroa* feed on hemolymph, the bees experience physical and physiological damage, protein levels decrease, and development can be abnormal [14], [15]. One of the worst impacts of *Varroa* comes from its association with honey bee viruses – mites can vector many honey bee viruses and some viruses can replicate within the mite [16]–[20]. Untreated *Varroa*-infested colonies usually die after one to four years of mite infestation; however, there have been reports of untreated hives with mites surviving for up to six years [3], [21]–[26].

Although *Varroa* have been effectively controlled with several miticides, pesticide-resistant populations of mites have appeared [27]–[39]. Miticides have significant drawbacks because they are soluble in the wax combs of the hive and can leave chemical residues in honey and wax, and synergism between chemicals can have negative effects on bee health [2], [34], [40]–[45]. A more sustainable form of control is desirable and the beekeeping industry has already started to benefit from the recent development of stocks that show resistance to mites [46].

A few behavioral traits of bees have been shown to reduce *Varroa* populations. One important trait is *Varroa* sensitive hygiene (VSH). Broadly, hygiene in honey bees refers to the act of adult bees removing dead, diseased or parasitized brood from sealed cells [47], [48]. Hygiene has been improved by breeding for bees that effectively remove free-killed brood (FKB). High hygiene bees also remove more *Varroa* than less hygienic bees [49], [50]. VSH is a form of hygiene in which bees have heightened response to *Varroa*; greater frequencies of mites are removed by VSH bees than by FKB hygienic bees [51](Danka et al. unpub. obs.). Enhanced mite removal enables VSH bees to effectively slow growth of the *Varroa* population in a colony [51]–[54]. VSH has a significant heritable component as evidenced by the response of the trait to selection in a USDA breeding program [52], [53], [55]–[57]. Field studies have shown that bees with the VSH trait successfully reduce mite infestations while retaining performance in traits important to beekeepers [58]–[60].

When infested brood is exposed to bees that exhibit high levels of VSH for one week, the mite reproduction decreases [52]. Immature mites may be killed due to uncapping and removal behavior [61]. When an infested pupa is removed from the colony, the adult female mite (and offspring) may be removed along with the pupa. If the adult female mites survive the removal of the host pupae, they usually attach to the bee that is removing the brood [62] but can also roam freely on the comb, where they are exposed to grooming behavior and can be detected and damaged via biting by the bees [49], [63]. It has also been suggested that mites which are removed with pre-pupae and pupae are not likely to produce viable offspring if they invade new brood cells too soon after such events [64]. Thus, the effectiveness of VSH on reducing mite reproduction is due partly to interference with reproduction, and in part to the risks the mite faces once it is out of the safety of the brood cell [65].

Here, we investigate the genetic architecture of VSH. A companion paper takes a similar approach to study mite-grooming behavior, the other behavior that affects mite population growth [66], [67]. The objective of the current study was to use quantitative trait loci (QTL) mapping on a genome-wide scale to look for segregating chromosomal regions for the VSH trait. Current selection relies on colony-level measurements of VSH; for example, observing a reduction in the level of mite infestation in brood or measuring reproductive success of individual mites in brood cells [55], [68]. Identifying the genes involved would assist in the understanding of the genetics and neurobiology of behaviors that confer mite resistance, as well as provide more efficient tools for selective breeding. Here we report progress towards that goal using a high-resolution genetic map integrated with the genomic sequence of the honey bee.

## Results

**Figure 1:**
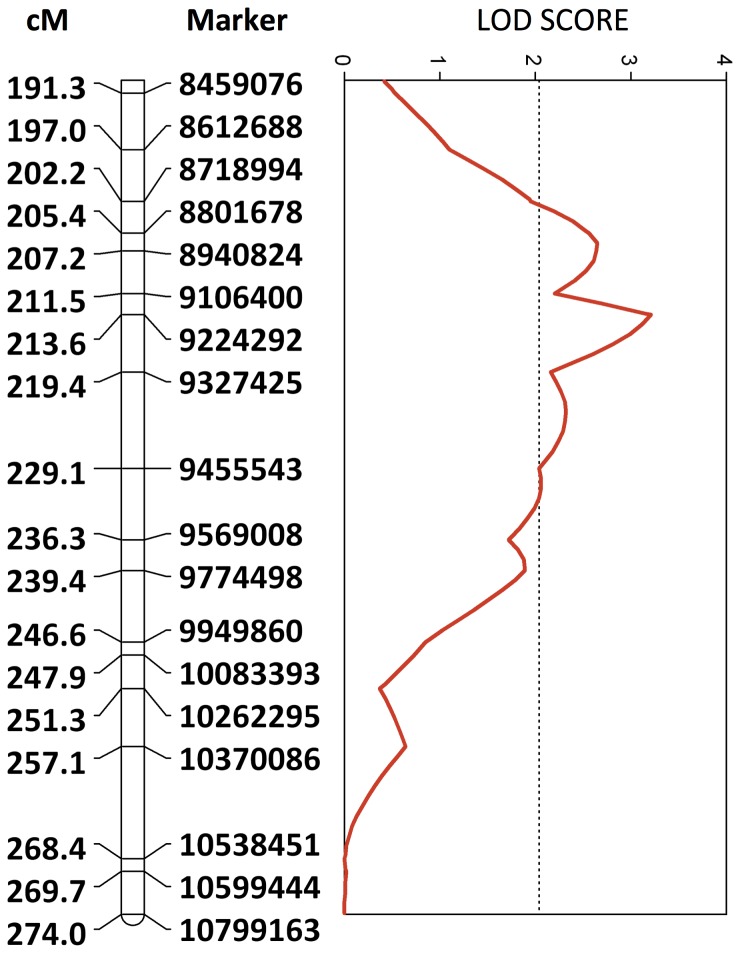
QTL location on map of chromosome 9. The physical location in base pairs of SNP probes in the honey bee genome assembly (Amel 4.0) is indicated to the right of the bar. Numbers to the left of the bar are distances in centimorgans (cM). The dotted line indicates the chromosome-wide empirical significance threshold of 0.05 as determined by 1000 permutations of phenotype data.

The genotypes for 1,340 informative SNPs were used to construct a high-resolution genetic map and to compare genotypes of individuals that performed VSH behavior (uncapping cells or removing infested pupae, n = 127) to those that did not (n = 111). The use of the Illumina GoldenGate assay provides high call rates and accuracy in calling SNP genotypes [69]. The high average recombination rate across the genome (whole genome: 22.6 cM/Mb, chromosome 9: 35.23 cM/Mb, chromosome 1: 26.144 cM/Mb) was similar to previous estimates [70]–[72]. Interval mapping analysis identified a LOD peak of 3.21 on chromosome 9 (Fig. 1). Permutation tests indicated that this QTL is not significant with the genome-wide threshold for p<0.05, however, it is does surpass the chromosome-wide threshold for p<0.05 (1000 iterations and p<0.05 thresholds: genome-wide  = 3.41, chromosome-wide  = 2.04) and is above the widely used theoretical threshold of 3.0 [71], [73]–[75]. On average, individuals that were homozygous for the VSH allele were more likely to be individuals who were observed exhibiting VSH behavior. This QTL explains 6.1% of the variance observed and had an effect size of 0.248408. The LOD-1.5 confidence interval spanned about 1.1 Mb of physical distance. There were 63 candidate genes identified in this region (Table S1). Two genes were particularly interesting given the association between general hygienic behavior and odors (Table 1) [76]–[79]: 1) *no receptor potential A2*, which is associated with vision and olfaction in *Drosophila*; and 2) *dop3*, a D2-like dopamine receptor, which has been shown to be involved in aversive olfactory learning and memory in *Drosophila* [80]–[82], crickets [83], and honey bees [84]–[86].

**Table 1:** Candidate genes involved in neurological signaling or regulation in QTL region on chromosome 9.

Honey beegene ID	*Drosophila*homolog ID	Predictions from Blast	Putative function
GB14619	CG3620	similar to no receptor potential A CG3620-PD, isoform D	phosphatidlyinositol phospholipase C activity; vision, olfaction
GB14561	CG33517	Dop3 D2-like dopamine receptor	aversive olfactory learning
GB15650		similar to dpr6 CG14162-PA	defective proboscis extension response; sensory perception
GB16925		similar to longitudinals lacking protein,isoform G	putative transcription factor for axon growth and guidance in the CNS and PNS
GB15048		similar to zinc finger protein 595;longitudinals lacking protein,isoform G-like	putative transcription factor for axon growth and guidance in the CNS and PNS
GB13523		similar to zinc finger protein 808-like	development of supraesophageal ganglion and ocelli; may promote appendage formation
GB10996		ATM interactor-like;longitudinals lacking protein,isoforms A/B/D/L	transcription regulation
GB10458		hypothetical protein LOC724938;longitudinals lacking protein,isoforms A/B/D/L	transcription regulation
GB12094, GB12494	CG12052	longitudinals lacking protein,isoforms A/B/D/L	transcription regulation
GB17677		hypothetical protein LOC100578231;longitudinals lacking protein,isoforms A/B/D/L	transcription regulation
GB14763		similar to zinc finger protein 407-like	transcription regulation
GB14706	CG7471	histone deacetylase Rpd3 isoform1Hist_deacetyl superfamily	transcription regulation
GB17640	CG2368	pipsqueak BTB superfamily	chromatin silencing; olfactory behavior
GB12634	CG12608	p21-activated protein kinase-interacting protein1-like; WD40 superfamily	signal transduction; pre-mRNA processing, cytoskeleton assembly
GB19232	CG17221	reticulon-4-interacting protein 1, mitochondrial-likeisoform 1; MDR superfamily; AdoMet_MTases superfamily	mushroom body development
GB11986, GB10237	CG5406	protein still life, isoform SIF type 1-like, partial;PH-like superfamily;UBQ superfamily,PDZ & RhoGEF superfamilies	signal transduction, regulation of synapse structure and activity
GB10808	CG3894	neuralized-like protein 2-like isoform 1;neutralized superfamily	signal transduction; myofiber differentiation and maturation
GB12006, GB16984		nicotinic acetylcholine receptorbeta2 subunit and alpha9 subunit;neur_chan_LBD superfamily	neurotransmitter-gated ion-channel ligand binding domain; ion transport
GB12219	CG3889	low quality protein: COP9;signalosome complex subunit 1;PCI superfamily	cell differentiation/specification; G-protein pathway suppressor 1
GB12004	CG2275	transcription factor AP-1;Jun superfamily;bZIP_1 superfamily	Jun-like transcription factor

A LOD peak of 1.95 was identified on chromosome 1, however, this QTL is only suggestive since it falls below both the genome-wide and chromosome-wide thresholds for significance (3.41 and 2.5, respectively). The percentage of observed variance explained by this QTL is 3.9% and the effect size is 0.196857. The LOD-1.5 confidence interval spanned approximately 2.0 Mb and contained 37 candidate genes, including a putative odorant receptor, a G-protein coupled receptor, and a protein that is a homolog of synaptic vesicle glycoprotein 2C (Tables 2 and S2).

**Table 2:** Candidate genes involved in neurological signaling or regulation in QTL region on chromosome 1.

Honey beegene ID	Drosophila homolog ID	Predictions from Blast	Putative function
GB19123	CG7497	prostaglandin E2 receptor EP4 subtype-like	regulation of Rhoprotein signal transduction
GB10077	CG16801	photoreceptor-specific nuclear receptor	transcription regulation
GB16999	CG31096	leucine rich repeat G protein coupled receptor	G-protein coupled receptor activity
GB18179	CG15302	putative odorant receptor 9a	olfaction, G-protein coupled receptor
GB10277	CG4898	tropomyosin-1; hypothetical protein LOC408583 isoform 1	muscle contraction; dendrite morphogenesis; lamellipodium assembly
GB17608	CG4898	tropomyosin-1	muscle contraction; dendrite morphogenesis; lamellipodium assembly
GB17660	CG4898	hypothetical protein LOC408583 isoform 1; tropomyosin	muscle contraction; dendrite morphogenesis; lamellipodium assembly
GB11694		hypothetical protein LOC100577365; segmentation polarity homeobox protein engrailed	compartment pattern specification; neuroblast fate determination
GB15566	CG9015	segmentation polarity homeobox protein engrailed	compartment pattern specification; neuroblast fate determination
GB18087	CG8759	nascent polypeptide-associated complex subunit alpha-like isoform 1	neurogenesis; oogenesis
GB14179		hypothetical protein LOC100577522; defective proboscis extension response, putative	defective proboscis extension response; sensory perception of chemical stimulus

## Discussion

We used genotyping arrays to analyze genotypes for 1340 SNPs in a set of 238 individuals to make a high-density QTL map for VSH-based resistance to *Varroa*. Six putative QTL influencing hygiene against FKB were previousy identified [87], but we do not see evidence of any of the same QTL in our study. This is despite other studies having shown that FKB hygiene confers some resistance to *Varroa* [25], [49], and that VSH or VSH-derived bees exhibit high FKB hygiene [65]. Oxley et al. 2010 [87] identified a QTL associated with FKB uncapping behavior on chromosome 9. The nearest marker reported falls outside of the confidence interval for the VSH-related QTL on chromosome 9 we found, and the exact position of the QTL reported for FKB hygiene is uncertain because of low marker density. This suggests that either different QTL are involved in VSH and FKB hygienic behavior, or that differences in the particular populations of bees we tested did not allow us to detect overlap of QTL intervals. In addition, proteomic profiling of honey bee antennae also showed no apparent overlap in peptide signatures between VSH bees and bees with FKB hygiene [88].

Differentially expressed genes between bees exhibiting high and low VSH were identified with microarrays [89]. The high VSH stocks were from the same general population that we used for this QTL study. The microarrays revealed 39 genes that were differentially regulated in the brains of 14-day-old worker bees of low- and high-VSH lines. The results did not fit the hypothesis that differences in VSH behavior were caused by differences in sensitivity to particular olfactory stimuli, although among the 39 genes were three that may be involved in olfaction (a putative odorant binding protein Est65A, *arrestin 2* and *Antdh* homologs). In contrast, the candidate genes we identified in our QTL mapping do show a possible connection between olfactory sensitivity and VSH and show no overlap with the differentially expressed genes in the microarray study. Linkage analyses have at least one advantage over microarray studies for identifying causal variation for traits – they directly tie the inheritance of genomic regions with the trait. Therefore if our QTL are confirmed in independent crosses, we can be confident that genes in the QTL regions are responsible for at least 10% of the differences in the trait measured. The candidate genes in our study may influence the expression of genes identified in the microarray study. It is also possible that although both studies used bees from the same USDA breeding program, different genes were segregating in each study, especially since the studies used different low lines. Brain tissue was used in the microarray study, but that may not be the only relevant tissue for VSH since olfaction starts with the antennae. Additionally, the studies differ in that the array study compared samples from colonies, not individuals, exhibiting different levels of VSH, whereas in our study, the comparisons were made between individuals that were observed performing the behavior.

The candidate genes identified by our study include no receptor potential A (*norpA*), a putative olfactory receptor, and a dopamine receptor. In addition to these candidates, there were genes in the QTL region with homology to *Dmel/dpr*, defective proboscis response, which are believed to be involved in chemosensory perception. *norpA* encodes a phospholipase C that is associated with vision in *D. melanogaster* [90]–[92], but also has been shown to affect olfaction; mutants defective in *norpA* exhibited impaired olfactory capabilities [93]. Phospholipase C has been documented in the homogenate of pheromone-sensitive sensilla of the silk moth, *Antheraea polyphemus* [94], and has been suggested as having a role in olfactory signal transduction in another moth, *Spodoptera littoralis* [95].

Dopamine (DA) is a catecholamine neurotransmitter and neuromodulator that is involved in behavior, cognition, learning, and memory in both vertebrates and invertebrates. In insects, appetitive learning is reinforced with octopamine and aversive learning is reinforced through a dopamine circuit. In *Drosophila*, dopamine-receptor-mutant larvae show impaired aversive learning [96], and blocking DA neurons in adults leaves the flies unable to learn to associate an odor stimulus with a punishment [82]. Similarly, in honey bees, the use of DA receptor antagonists blocked aversive learning (exhibited by the extension of a bee’s sting in response to an odor that it was trained to associate with electric shock) [86]. Honey bees have three dopamine receptors (see [97] for review); *dop3* is a D2-like dopamine receptor that is widely expressed in the honey bee brain, but shows noticeably different expression from that of *dop1* and *dop2* [98]. The distribution of *dop3* mRNA in cells around the optic and antennal lobes of the honey bee brain also suggests that this D2-like dopamine receptor is involved in processing sensory information [84], [98].

Olfactory cues have been shown to mediate general hygienic behavior [76]–[79], but the role of odor as a stimulus for hygiene by honey bees against *Varroa* is unclear. Earlier work suggested that the odor of the mite itself is probably not an important cue to *A. mellifera* [99]. Schöning et al. 2012 [100], however, suggested that bees recognize damaged brood by olfactory cues. The odor profile of brood parasitized by mites with high potential to transmit deformed wing virus (DWV) differed from the odor profile of brood parasitized by mites with low potential to transmit DWV. Hygienic bees preferentially removed pupae infested with mites with a high potential to induce damaging DWV infections, which are more likely to cause deformities and death. Our results support an association between genes involved in olfaction and VSH; however, we cannot rule out the possibility that other (non-olfactory) genes in our QTL regions modulate VSH.

Further work to identify the genes underlying this trait and then utilizing them as diagnostic tools for selective breeding could be valuable for beekeeping. Our mapping study will be followed with studies to analyze differential expression of candidate genes, gene function and association with VSH using gene knockdowns, and sequence differences between alleles. In order to use SNP markers for marker-assisted selection (MAS), it probably will be necessary to have SNP markers within the causal genes because of the high recombination rate of the honey bee genome. MAS may also allow for simultaneous selection and breeding for multiple traits, such as VSH, grooming behavior and physiological resistance to *Varroa* [67], [101]. If these technical challenges are met and useful markers are developed, MAS may speed selection by targeting sequences of specific genes in potential breeder queens and drones.

## Materials and Methods

### Ethics Statement

No permits were required to conduct the field research or genotyping analyses. The crosses and field research were conducted at the USDA-ARS Honey Bee Breeding, Genetics and Physiology Laboratory in Baton Rouge, LA, which is established and maintained to conduct apicultural research and bee breeding. Genotyping was performed in the Purdue core genomics facility in accordance with university and federal biosafety regulations.

### Source of Worker Bees

Queens that produced colonies with either high or low expression of *Varroa* sensitive hygiene (VSH) were chosen as parents for the production of experimental colonies. The high VSH line was chosen from an ongoing USDA selection program. The high VSH queen produced a colony that removed 85–95% of mite-infested pupae during assays in which a comb of infested capped prepupae was placed into the broodnest for a 1-week period (method as in [68]), while the low VSH queen produced a colony that removed no more than 15% of the mite-infested pupae in similar assays. Fifteen daughter queens from the high VSH line were each mated to a single drone from the low VSH parent using instrumental insemination. The colonies containing F1 workers produced by these queens were evaluated for VSH activity and the colony that had removed the highest percentage of mite-infested pupae (62%, mean of 15 colonies  = 40.6%) was used to produce 17 F1 daughter queens. Each F_1_ daughter queen was backcrossed to a single drone from the high VSH parent, and each colony was evaluated for VSH activity about 7–8 weeks later (mean  = 59.0% removal). The colony with the highest removal (83%) of mite-infested pupae, colony A, was used as the source of worker bees that were evaluated for QTL analyses. The drones and queens used to make all generations of crosses were frozen and saved for SNP genotyping. All breeding and behavioral studies were conducted at the USDA, ARS Honey Bee Breeding, Genetics and Physiology Laboratory in Baton Rouge, LA.

### Behavioral Studies of Worker Bees

Workers from colony A were classified as hygienic or non-hygienic by direct observation of their behavior when exposed to a comb of highly mite-infested pupae during a 45 minute period. Multiple tests over several weeks were needed to obtain enough workers to perform QTL analyses. Each week for 5 weeks, 300–500 newly emerged workers were individually marked by gluing a small plastic numbered disc to the thorax (E. H. Thorne, Ltd., Lincolnshire, UK); tags were also marked with paint to create enough unique combinations so that each individual bee could be identified. Workers were returned to their colony shortly after being tagged.

Behavioral testing began during the 3^rd^ week when the oldest tagged workers were 15–18 days old, which corresponds to the optimal age for expression of hygienic behavior [102], and continued until 125 workers were identified as non-hygienic and another 125 workers were identified as hygienic (through the 5^th^ week). Each test began when a comb containing mite-infested capped brood was inserted into the center of the broodnest of colony A. Combs were taken from heavily infested colonies and were chosen only if they had >100 square inches of capped brood, 15–20% *Varroa* infestation levels, and the pupae were predominantly in the white-eyed to pink-eyed stages [55]. The mite-infested comb was left in the center of the broodnest for 15 minutes; afterwards, it was removed carefully with adhering bees to an observation hive kept within a warm room. Two people conducted the behavioral observations, one on each side of the comb. Workers were identified as hygienic if they were observed (1) perforating the wax capping of the cell of a pupa, (2) enlarging the hole of an already perforated cell cap, or (3) removing a pupa from a fully uncapped brood cell. Workers were only sampled if they engaged in these behaviors for >2 minutes, and if the targeted brood cell was infested by *Varroa*. To determine if a brood cell was infested, a numbered pin was placed next to the brood cell that was manipulated at the moment that each worker bee was sampled. At the end of the test, the remaining worker bees were gently shaken and brushed from the combs, and each manipulated brood cell was examined under a stereomicroscope for the presence of *Varroa*. Workers were eliminated from the pool of hygienic bees if their hygienic responses were being directed toward cells infested by larvae of the greater wax moth (*Galleria mellonella*) or the small hive beetle (*Athena tumida*), or if the pupa was not mite-infested. Non-hygienic workers were identified as workers from the same age cohort as the hygienic workers that did not attempt hygiene during the 15 minutes of direct observation. Most non-hygienic workers were observed standing or walking over brood or engaging in trophallaxis with no attempts to engage in uncapping or removal behavior.

Each hygienic and non-hygienic worker was grabbed from the comb surface using soft forceps and quickly inserted into a plastic vile, which was flash frozen in liquid nitrogen. All samples were stored at −80°C until needed.

### Genotyping and QTL Mapping

The DNA of the F1 queen was extracted using the Qiagen DNeasy Blood and Tissue Kit (Qiagen Inc., Valencia, CA) and was sequenced using the ABI SOLiD platform (Life Technologies Corp., Carlsbad, CA). We identified SNPs and designed probes for 1,536 genome-wide SNPs. These probes were used to analyze the genomic DNA of worker bees from the backcross family.

DNA was extracted from 240 individual worker abdomens using Qiagen DNeasy Blood and Tissue Kits. DNA was quantified with a fluorometer (Turner BioSystems, Sunnyvale, CA) and all samples diluted to 50 ng/µl. Genotyping was performed using the Illumina GoldenGate Assay with 250 ng of DNA per individual. Details of the assay can be found at the Illumina website (Illumina, Inc., San Diego, CA, www.illumina.com), but briefly, DNA is fragmented and activated for binding to paramagnetic particles, then hybridized with allele-specific and locus-specific oligonucleotides. The last 3′nucleotide of the allele-specific nucleotide is at the SNP. Extension past the SNP and ligation to the locus-specific oligo follow, giving rise to full-length joined products that serve as templates for PCR with universal primers and dye-labeled allele-specific primers. The dye-labeled PCR products were hybridized to the genotyping array matrix using a complementary address sequence present in the locus-specific primer. The fluorescence signals were read by the BeadArray Reader and analyzed by GenomeStudio software for semi-automated genotype clustering and calling (Illumina, Inc). Probes that had low call rates or were not polymorphic were removed from the data set (216 SNPs).

SNP markers were assembled into linkage groups using JoinMap 4.0 software [103], [104]. The marker orders were obtained by maximum likelihood analysis. Linkage distances between markers were estimated using multipoint analyses and the Kosambi mapping function. Interval mapping was performed with MapQTL 5.0 software [105]. The phenotypes were coded as a binary trait (1 or 0, depending on whether individuals exhibited the behavior). This analysis is effectively interval mapping with the Chi-square statistic. The 1.5-LOD support intervals (which correspond roughly to the 95% confidence intervals) for the QTL positions were determined from the interval mapping LOD values [106] and candidate genes were identified. Sequences for the probes that fall within the 1.5-LOD intervals can be found in Table S3. Genome-wide permutation tests were performed in MapQTL 5.0 to calculate the empirical significance thresholds to identify significant and suggestive QTL [107].

## Supporting Information

Table S1 **Complete list of candidate genes for QTL region on chromosome 9.**
(DOCX)

Table S2 **Complete list of candidate genes for QTL region on chromosome 1.**
(DOCX)

Table S3 **Probe sequences used for genotyping that fall within the 1.5-LOD support interval.**
(DOCX)
