# Supplementary material for: High-Resolution Linkage Analyses to Identify Genes That Influence Varroa Sensitive Hygiene Behavior in Honey Bees
Source: PLoS One. 2012 Nov 2;7(11):e48276. doi: 10.1371/journal.pone.0048276 (PMC3487727; doi:10.1371/journal.pone.0048276)
Supplement: Table S1 — Complete list of candidate genes for QTL region on chromosome 9. (DOCX) [file pone.0048276.s001.docx]

| **Honey bee gene ID** | ***Drosophila* homolog ID** | **Predictions from Blast** | **Putative function** |
| --- | --- | --- | --- |
| GB30419 |  | cuticular protein 11 |  |
| GB30420 |  | similar to hypothetical protein LOC725710 |  |
| GB18182 |  | similar to methyltransferase-like protein6 |  |
| GB14905 |  | hypothetical protein EAG_14606 |  |
| GB10410 |  | hypothetical protein EAI_08985 |  |
| GB17322 |  | hypothetical protein LOC725903 |  |
| GB13274 | CG7031 | hypothetical protein LOC725903 |  |
| GB10653 |  | hypothetical protein LOC726031 |  |
| GB14619 | CG3620 | similar to no receptor potential A CG3620-PD, isoform D | phosphatidlyinositol phospholipase C activity; visual transduction; olfaction |
| GB16013 |  | hypothetical protein LOC726078 |  |
| GB14561 | CG33517 | Dop3 D2-like dopamine receptor | aversive olfactory learning |
| GB15650 |  | basement membrane-specific heparin sulfate proteoglycan core protein-like; similar to dpr6 CG14162-PA | defective proboscis extension response; sensory perception of chemical stimulus |
| GB12836 |  | hypothetical protein LOC724395 |  |
| GB18280 |  | limbic system-associated membrane protein-like |  |
| GB14580 |  | histone-lysine N-methyltransferase SETMAR-like |  |
| GB15156 |  | zinc finger protein 26-like |  |
| GB11073 |  | uncharacterized protein LOC100864777 |  |
| GB16925 |  | similar to longitudinals lacking protein isoform G | putative transcription factor required for axon growth and guidance in the CNS and PNS |
| GB15048 |  | similar to zinc finger protein 595; longitudinals lacking protein isoform G-like | putative transcription factor required for axon growth and guidance in the CNS and PNS |
| GB15003 |  | similar to zinc finger protein 676 |  |
| GB13523 |  | similar to zinc finger protein 808-like | required for development of the supraesophageal ganglion and ocelli; may promote appendage formation |
| GB10996 |  | ATM interactor-like; longitudinals lacking protein, isoforms A/B/D/L | transcription regulation |
| GB10458 |  | hypothetical protein LOC724938; longitudinals lacking protein, isoforms A/B/D/L | transcription regulation |
| GB12494 |  | longitudinals lacking protein, isoforms A/B/D/L | transcription regulation |
| GB17677 |  | hypothetical protein LOC100578231; longitudinals lacking protein, isoforms A/B/D/L | transcription regulation |
| GB12094 | CG12052 | longitudinals lacking protein, isoforms A/B/D/L | transcription regulation |
| GB14763 |  | similar to zinc finger protein 407-like | transcription regulation |
| GB14706 | CG7471 | histone deacetylase Rpd3 isoform 1Hist_deacetyl superfamily | transcription regulation |
| GB17640 | CG2368 | pipsqueak; BTB superfamily | chromatin silencing; olfactory behavior |
| GB16277 | CG34015 | histidine triad nucleotide-binding protein 3-like; aprataxin_related; HIT_like superfamily | carbohydrate transportation and metabolism |
| GB10793 |  | mitochondrial enolase superfamily member 1-like; MR_MLE_N superfamily; enolase_like superfamily |  |
| GB18583 | CG30394 | putative sodium-coupled neutral amino acid transporter 10-like; Aa_trans superfamily | transmembrane amino acid transporter protein |
| GB19520 | CG11658 | f-box only protein 32-like |  |
| GB16093 | CG4338 | UPF0293 protein C16orf42-like; RLI superfamily; DUF367 superfamily | RNA L inhibitor superfamily -plays a role in the anti-viral and anti-proliferative activities of interferons |
| GB16097 |  | hypothetical protein LOC725634 |  |
| GB14091 |  | hypothetical protein LOC409232 |  |
| GB14020 |  | dipeptidase 1-like isoform 2; metallo-dependent_hydrolases superfamily | amino acid transport and metabolism |
| GB11859 |  | dipeptidase 1-like isoform 2;metallo-dependent_hydrolases superfamily | amino acid transport and metabolism |
| GB13209 |  | endothelin-converting enzyme 1-like; GluZincin superfamily; peptidase_M13_N superfamily | proteolysis |
| GB12634 | CG12608 | p21-activated protein kinase-interacting protein 1-like; WD40 superfamily | signal transduction, pre-mRNA processing and cytoskeleton assembly |
| GB11883 | CG3430 | mini-chromosome maintenance complex-binding protein, isoform 2 | DNA replication |
| GB30249 |  | NF-kappa-B inhibitor cactus-like, (LOC725766) |  |
| GB30250 |  | NF-kappa-B inhibitor cactus 3, transcript variant 2 (cact3) |  |
| GB13565 |  | inositol hexakisphosphate kinase 2-like DUF2475 superfamily | protein phosphorylation, phosphatidylinositol metabolic processing |
| GB17383 | CG10082 | inositol hexakisphosphate kinase 2-like DUF2475 superfamily | protein phosphorylation, phosphatidylinositol metabolic processing |
| GB12719 | CG7441 | tryptophanyl-tRNA synthetase, mitochondrial-like; Nt_trans superfamily | tryptophanyl-tRNA aminoacylation |
| GB19232 | CG17221 | reticulon-4-interacting protein 1, mitochondrial-like isoform 1; MDR superfamily; AdoMet_MTases superfamily | mushroom body development |
| GB11986 |  | protein still life, isoform SIF type 1-like, partial; PH-like superfamily; UBQ superfamily, PDZ superfamily; RhoGEF superfamily | signal transduction, regulation of synapse structure and activity |
| GB10237 | CG5406 | protein still life, isoform SIF type 1-like, partial; PH-like superfamily; UBQ superfamily, PDZ superfamily; RhoGEF superfamily | signal transduction, regulation of synapse structure and activity |
| GB14749 |  | niemann-Pick C1 protein-like; sterol sensing superfamily | sterol metabolism |
| GB14991 | CG9047 | sodium/potassium-transporting ATPase subunit beta-1-interacting protein; hypothetical protein LOC552749; NKAIN superfamily | regulation of salt ion transport |
| GB16408 | CG8772 | glutaminase kidney isoform, mitochondrial isoform 1; glutaminase superfamily | morphogenesis; glutamine catabolic process |
| GB10808 | CG3894 | neuralized-like protein 2-like isoform 1; neutralized superfamily | signal transduction; myofiber differentiation and maturation |
| GB12006 |  | nicotinic acetylcholine receptor beta2 subunit and alpha9 subunit; neur_chan_LBD superfamily | neurotransmitter-gated ion-channel ligand binding domain; ion transport |
| GB16984 |  | nicotinic acetylcholine receptor beta2 subunit and alpha9 subunit; neur_chan_LBD superfamily | neurotransmitter-gated ion-channel ligand binding domain; ion transport |
| GB10568 | CG1271 | putative glycerol kinase 5-like isoform 2; putative glycerol kinase 5-like isoform 1; FGGY_N superfamily; FGGY_C superfamily | glycerol metabolism |
| GB12681 | CG11140 | aldehyde dehydrogenase family 3 member B1; ALDH-SF superfamily | detoxification; cellular aldehyde metabolic process |
| GB12219 | CG3889 | low quality protein: COP9 signalosome complex subunit 1 isoform 1; PCI superfamily | cell differentiation and specification; G-protein pathway suppressor 1 |
| GB14290 |  | hypothetical protein LOC726215 |  |
| GB19672 | CG6495 | hypothetical protein LOC552693 isoform 2; low-density lipoprotein receptor-related protein 11 | endocytosis |
| GB11386 | CG17271 | multiple coagulation factor deficiency protein 2 homolog; EFh superfamily | calcium sensing and calcium signal modulation |
| GB15720 |  | hypothetical protein LOC552674 |  |
| GB12004 | CG2275 | transcription factor AP-1; Jun superfamily; bZIP_1 superfamily | Jun-like transcription factor |
