# Supplementary material for: High-Resolution Linkage Analyses to Identify Genes That Influence Varroa Sensitive Hygiene Behavior in Honey Bees
Source: PLoS One. 2012 Nov 2;7(11):e48276. doi: 10.1371/journal.pone.0048276 (PMC3487727; doi:10.1371/journal.pone.0048276)
Supplement: Table S2 — Complete list of candidate genes for QTL region on chromosome 1. (DOCX) [file pone.0048276.s002.docx]

| **Honey bee gene ID** | **Drosophila homolog ID** | **Predictions from Blast** | **Putative function** |
| --- | --- | --- | --- |
| GB13163 | CG1650 | hypothetical protein LOC725495; homeobox protein GBX-1 | transcription regulation |
| GB12769 | CG1232 | protein tipE-like | enhances para sodium channel function; protects against heat-induced lethality |
| GB19011 | CG18676 | hypothetical protein LOC100577273 |  |
| GB10333 | CG15004 | protein tipE-like |  |
| GB17990 | CG15003 | hypothetical protein LOC100577239 |  |
| GB16023 | CG18675 | uncharacterized protein C21orf59 homolog |  |
| GB16181 | CG34389 | hypothetical protein LOC410706; StAR-related lipid transfer protein 13 | Steroid biosynthetic process |
| GB13413 | CG7713 | hypothetical protein LOC551206 |  |
| GB12299 | CG43102 | hypothetical protein LOC413562 |  |
| GB19123 | CG7497 | prostaglandin E2 receptor EP4 subtype-like | G-protein coupled receptor activity |
| GB11311 | CG7549 | hypothetical protein LOC413560 |  |
| GB10049 | CG6232 | thrombospondin type-1 domain-containing protein 4-like | metalloendopeptidase activity |
| GB10077 | CG16801 | photoreceptor-specific nuclear receptor | transcription regulation |
| GB17865 | CG5934 | short coiled-coil protein B-like | mitotic spindle assembly; regulates microtubules severing |
| GB14157 |  | heparin-binding growth factor 1-like; hypothetical protein LOC551529 | growth factor activity |
| GB12314 | CG31534 | uncharacterized protein LOC100866274 |  |
| GB19573 | CG31531 | uncharacterized protein LOC100877226 |  |
| GB16999 | CG31096 | leucine rich repeat G protein coupled receptor | G-protein coupled receptor activity |
| GB18179 | CG15302 | putative odorant receptor 9a | olfaction, G-protein coupled receptor |
| GB11344 | CG9854 | poly(A) polymerase gamma | RNA polyadenylation |
| GB15278 | CG42402 | hypothetical protein LOC724835 |  |
| GB17666 | CG12806 | protein tipE |  |
| GB10277 | CG4898 | tropomyosin-1; hypothetical protein LOC408583 isoform 1 | muscle contraction; dendrite morphogenesis; lamellipodium assembly |
| GB17608 | CG4898 | tropomyosin-1 | muscle contraction; dendrite morphogenesis; lamellipodium assembly |
| GB17660 | CG4898 | hypothetical protein LOC408583 isoform 1; tropomyosin | muscle contraction; dendrite morphogenesis; lamellipodium assembly |
| GB13779 | CG3682 | phosphatidylinositol-4-phosphate 5-kinase type-1 alpha-like isoform 2hypothetical protein LOC724991 | phosphatidylinositol phosphate kinase activity |
| GB14379 | CG15020 | hypothetical protein LOC725078 |  |
| GB11694 |  | hypothetical protein LOC100577365; segmentation polarity homeobox protein engrailed | compartment pattern specification; neuroblast fate determination |
| GB15566 | CG9015 | segmentation polarity homeobox protein engrailed | compartment pattern specification; neuroblast fate determination |
| GB18087 | CG8759 | nascent polypeptide-associated complex subunit alpha-like isoform 1 | may promote appropriate targeting of ribosome-nascent polypeptide complexes; development |
| GB19383 | CG8735 | protein lunapark-B-like |  |
| GB11159 | CG10726 | condensin complex subunit 2-like | regulatory subunit of condensing complex, which probably introduces positive supercoils into relaxed DNA; DNA condensation |
| GB11992 | CG4065 | n-alpha-acetyltransferase 35, NatC auxiliary subunit | embryonic cell proliferation and survival |
| GB15897 | CG13855 | IQ and ubiquitin-like domain-containing protein-like |  |
| GB18990 |  | IQ and ubiquitin-like domain-containing protein-like |  |
| GB18958 |  | sodium channel protein Nach | tracheal liquid clearance; sodium transport |
| GB14179 |  | hypothetical protein LOC100577522; defective proboscis extension response, putative | defective proboscis extension response; sensory perception of chemical stimulus |
