# Supplementary material for: High-Resolution Linkage Analyses to Identify Genes That Influence Varroa Sensitive Hygiene Behavior in Honey Bees
Source: PLoS One. 2012 Nov 2;7(11):e48276. doi: 10.1371/journal.pone.0048276 (PMC3487727; doi:10.1371/journal.pone.0048276)
Supplement: Table S3 — Probe sequences used for genotyping that fall within the 1.5-LOD support interval. (DOCX) [file pone.0048276.s003.docx]

| **Probe name** | **Sequence** |
| --- | --- |
| >1_17408194 | GTTATCAACCGTTGAAATAAACTTCGTGTTAATTTTAAGGGGAAGGAGGAGAGAATTCGC[A/G]TTAACGAATCTCGAGGGCGACGATTTCCGCGTTTCTGGCGAATTCTGTACGCCCAGAAAT |
| >1_17484642 | TGCTCTTCGAGCAAATATGCATAAAGGTACGTGGCGAAAATACTTCTCGCGTAACAAGCG[T/C]CTTTTGCATTCCCCTGCAACCGTTTCGACCGAGTCGTCGACATTGTCTTTGGCCGTGATA |
| >1_17577515 | GTTGAATCATTATCACCCCTCCCCTTTTTTTATAAGCTATGACAAACTCACCCTCTAATG[T/C]CTCACGACGGACTAACACTGGAAAGTTTCCACTTCTCGCGAGCAATAACTCGTCCCATTG |
| >1_17928878 | AAAACCGATGTCACCGCTTCTACCAGGAGTTCTAATACCGACGAGGTATCGAGCGAAAAC[A/G]GAGAAGCGAGGAGATTCGACGAACGTGGTTTAGTCTTTCCCTCGTCTCTCGGGAAGGAAA |
| >1_18094359 | GGGCCCCTTGCACTTGCGATTCCAGATAATCATCTTCCTCCAAGATTCACCTCATCCTCC[A/G]TAATCTCAGAAAACTACTTTACGAAACTACTTTATACTTATATTCCACGTCCTCGATCAA |
| >1_18214692 | AAAGTCAAAGTCTTATCTTAGTTATCAACCAAGGTTCGAAGAAATGACAGTAATTTTCTA[T/C]GAGGTGAAAGCGGAAATTCGCCATTTTTCATTTTTCTTCGTTCCCTGTCCTCTTTATCCC |
| >1_18318085 | AATGAGATCGATTTCTATTCCTCTCAACGAGTTCTTCTTTTTCAGACGACATTTTTATCC[A/G]TGTATAGTACCAAGATGTTCCATTTAATTCCTTTTTTAATTCGATTTTCAATCATCCATG |
| >1_18496051 | TCTCCATTAATGCTGTCACGTCACGTCACGTCACGCCTTCCAGGTGGCCGAGTGACCTGT[T/C]GTTGACAGCTTTTCTATCGTTGATGCTTATAAGATCGAAATTCACCCCTGTAAATTCACC |
| >1_18596602 | TTTAATGGCACTCGTCGCGCAACATTGTCGGCCAGAAACGACTGCTCGCACATTGTAGGG[A/G]AGTTTATGACACCGGCTGGAATCGGCAAAGAGGACAAGAAAATAAAACTCGTTAAGGATG |
| >1_18786427 | GTAATATAATCAAATCAATTGCATCCCTTGCTATCCATCGTTAATCAAAATTCAAACAGC[A/G]TGCAACCCCCTCCAAACGATAGATAGATTTCAATCTCACCGAAATATTTTCCCTCTATAA |
| >1_19029230 | GGGTTCGAATTCACAGGATGCTCGTTGTTCCTCGAGTTCCCACAGATCTTGATAATTCCA[T/C]GCGTGACGAGACATCGAAAATCGAACGACTTTTGGGTTCGCTAATCGAACGAGAACCGAG |
| >1_19187323 | GGTTAAACTTCATTCGCTCGTCGTGACACTCCTATTTGCATAAGACGTAAAGGAGGAGGA[A/G]GGAAAAGCGTGAAAATTGCGGAAATTCGGTGTGAAAGGCCGAAGGGTGGCCGCTCTCCCC |
| >1_19282063 | CAAAAAAAAGGGAATCGTGAAAGAAAACCGTATCGGACACGGTTTGTGACTAAGCAAGGG[A/G]TAATGAACGAAAAATTTCAAAGCGAGACGTAATGAAATATTGGAGAAATGTGGAAGACGC |
| >9_8718994 | CACTGAGGTTTTGAGGAGTTTGAAAGCTTTGGAGGATAAATACGTGATGGGAATGGTATC[T/C]TATGCTTCGTAATATAGCTTCGAAATATAGAAACCAGTTAATAACAGGAGAGATAATTTT |
| >9_8801678 | TTGGTCGTGAAAGCGGAAGAACGCGGAATGTCGTAATATAGCTTATCCCTGTTGATAGTC[T/C]CCGCCGCATTCGCTAAACGGCAGCCCGCTCGTAAAACTTCTCTCGAGCTTGAAGCAGCGT |
| >9_8940824 | ATTTCCTCCTAAACCCTTTTGAAACGTGAGTGGCGTACGTGCTGTTGGAAACTGATCTTG[T/C]GATCTTAACTCTCCCCCTTCCGAAACTCAAACTCGCGCGTTTTAACGATTATCGTTAACC |
| >9_9106400 | AAAACGTTAATGAATTCCTATAGAAATTTCTCACCTAGCCCCAGGAATGGTTGAATAATT[A/G]CGTGAATGAGTAATTACAGTGTACGGTTTCTCCGTCAACGAGAATGAGACAAAGTTACCC |
| >9_9224292 | CGGGTTTCCCTTCCCCGATGCTTCACAGGTTGCTAATTCATTATTGTTGCAACCGAGTTT[T/C]CCCAACTTTGTAAATACACACATACACGCGGGATGTTTCTCTGGAGGGGATAAGACACGC |
| >9_9327425 | TATCGATTTACTTTCGTTAAATTGTTCTCTATAACGATAATGATTTCCATCGTCGCAGGT[A/G]ACATGGATCAGAAGAAAGGACAGGCAATTACTGACGTTGGGGACGGGCACCCACGCCATC |
| >9_9455543 | GTGTATACGTATGCATGTTGTGTGTATGTATGTATGTACGTATACGTGTGTAGCAGGTAT[A/G]TGGATACAAACGACAAAAAACGGACTCGCGGTTGCCGTGAAAAGAGAGAGAGAAGAGGAG |
| >9_9569008 | ACCAATCTCGTGGACCGCGCTAAACTAGTTAAGCCGCGACGCCTAGCGTTGGATACGGAT[A/G]CCGCGGAAAGCAAACAAAGTTCGAGCAACATGTGTCGAGAAGAAAAATAATAATTCTGTC |
| >9_9774498 | CTCCGACACCGAGGAATCGTCTCAGAACATAGAGGATTTGTCCATATCCACGAATCCCAT[A/T]GAGGTGATGCAAAACAGTCCTGTAATCAGTTTCACGCAGTTGAACAGCGAGGATAAAGCG |
